# Fathers’ Sensitivity in Infancy and Externalizing Problems in Middle Childhood: The Role of Coparenting

**DOI:** 10.3389/fpsyg.2022.805188

**Published:** 2022-02-08

**Authors:** Deborah Jacobvitz, Ashleigh I. Aviles, Gabriela A. Aquino, Ziyu Tian, Shuqi Zhang, Nancy Hazen

**Affiliations:** Department of Human Development and Family Sciences, The University of Texas at Austin, Austin, TX, United States

**Keywords:** coparenting, family systems, fathers, caregiving, externalizing symptoms

## Abstract

The present study examined the role of father sensitivity and couple coparenting quality in the first 2 years of life in relation to the development of externalizing behavior problems in middle childhood, focusing on the unique role of fathers. In this study, 125 mothers, fathers, and their first-born children were followed from 8 months to age 7 years. Paternal sensitivity was rated when infants were 8 and 24 months old. Fathers were videotaped at home playing, feeding, and changing their 8-month-old infants’ clothes. They also were videotaped in a lab playing with their 24-month-olds and solving a variety of challenging tasks. At 24 months, competitive coparenting was assessed *via* videotaped triadic family interactions at home in which families participated in a variety of tasks (i.e., clothes change, eating a snack together and solving tasks). Teachers rated externalizing behavior problems when the children were age 7. Continuity in paternal sensitivity was documented from 8 to 24 months, and paternal sensitivity at 8 months predicted externalizing behavior in middle childhood through father sensitivity at 24 months. Moreover, paternal sensitivity at 8 months predicted competitive coparenting which, in turn, forecast externalizing behavior problems in middle childhood, even after controlling for maternal sensitivity at 8 and 24 months. These findings highlight the unique role of paternal caregiving quality during the first year of life on couple coparenting and children’s subsequent development of externalizing problems and have implications for creating effective interventions to prevent children from developing externalizing disorders.

## Introduction

Externalizing behaviors in early and middle childhood include temper tantrums, defiant behavior, impulsivity, social maladjustment, and reduced tolerance for frustration (Murphy et al., 2017a). Further, these behavior problems increase the likelihood of alcoholism, psychological disorders, drug abuse, and maladaptive relationships during adolescence and adulthood (Masten et al., 2005).

Identifying early antecedents of externalizing behaviors is important to help prevent these maladaptive behaviors from developing. Both maternal (Lorber and Egeland, 2009) and paternal (Rodrigues et al., 2021) sensitivity during the early years, defined as accurately perceiving and appropriately responding to the child’s emotional and cognitive signals (Ainsworth et al., 1978), forecast fewer later externalizing symptoms in childhood. Yet, research on father–infant interaction and its relation to child outcomes still lags far behind research on the effects of mother–infant interaction. One explanation for the lack of systematic and rigorous research on paternal caregiving is that the focus on fathers as economic providers has led to the view that they do not spend enough time with their children to affect their lives emotionally (Cabrera et al., 2018). There has been a surge in women’s labor force participation since the 1970s. As the gender gap in the share of the work force held by men and women has narrowed, the amount of time fathers spend interacting with their infants has increased three- to six-fold in Western countries (Bakermans-Kranenburg et al., 2019). Thus, it is important to understand the lasting effects of paternal sensitive caregiving during the first 2 years of life on children’s development.

Fathers’ sensitive caregiving might reduce infants’ risk for the later development of externalizing symptoms not only directly, but also indirectly, by affecting coparenting quality, which refers to the ways in which both parents work together to parent their child (McHale et al., 2001). Negative patterns of coparenting, particularly competitive coparenting in which parents undermine each other in front of the child have been linked to children’s later externalizing behavior (Teubert and Pinquart, 2010; Murphy et al., 2016). Thus, the goal of the present study is to examine the role of paternal sensitivity and coparenting quality in the first 2 years of life in the development of children’s externalizing behavior problems in middle childhood. We propose that the quality of care that fathers provide their infants at 8 months will relate to their development of externalizing problems in middle childhood through two pathways. (1) Fathers’ sensitive care will be stable from 8 to 24 months, and fathers’ sensitive caregiving at 24 months will predict lower child externalizing problems in middle childhood, and (2) Fathers’ sensitive care at 8 months will be associated with coparenting quality, which will, in turn, forecast externalizing problems in middle childhood.

This study will be one of the first to examine stability of paternal caregiving over the first 2 years of life. There is evidence of stability in paternal care from middle childhood to adolescence (Bureau et al., 2017), but less is known about stability of paternal care over the first 2 years. Maternal sensitivity has been shown to be stable from 10 to 12 months (Behrens et al., 2012) and greater stability in maternal sensitivity has been found from 15 to 24 months than 6 to 24 months (Dallaire and Weinraub, 2005). This is likely because dyadic reciprocity increases substantially at 8 months, when infants are more able to contribute meaningfully to the give-and-receive exchange (Feldman, 2010). For example, infants begin to communicate by pointing and gesturing, and, when upset, they can seek proximity to the caregiver by vocalizing and crawling to the parent (Jacobvitz et al., 1991). Based on these findings, we assessed paternal sensitivity when infants were 8 months of age to examine stability of paternal care from 8 to 24 months and the role of father sensitivity in coparenting quality and children’s later behavior problems.

### Father Sensitivity and Children’s Externalizing Behavior

Fathers’ sensitive interaction with their infants and young children has been theorized to play a unique role in the development of their children’s emotion regulation (Paquette, 2004; Hazen et al., 2010), which is critical for reduction of children’s externalizing behaviors. According to Grossmann and Grossmann (2020), fathers tend to prioritize exploration and stimulating play, such as rough-and-tumble play, when they interact with their infants and toddlers. This play may become overstimulating or even frightening to these young children, so that fathers need to calm them. One study found that fathers who were more sensitive while engaging in highly stimulating and potentially frightening play with their 8-month-old infants, compared to those who were less sensitive during this type of play, were more likely to have children who were better regulated at 24 months (Hazen et al., 2010). Similarly, fathers’ sensitive interaction with their toddlers during challenging, stimulating play was associated with their children’s attachment security during middle childhood, adolescence, and early adulthood (Grossmann and Grossmann, 2020). Thus, it is possible that fathers who engage in challenging play with their young children and can sensitively comfort them when they become overstimulated or frightened, may be scaffolding their ability to regulate their impulses to engage in externalizing behaviors. In contrast, fathers who continue to engage in stimulating play when their young children become upset may further dysregulate their children and exacerbate their externalizing behaviors (Hazen et al., 2010).

Few studies have examined associations between paternal sensitivity with young children and children’s later development of externalizing behavior. Two cross sectional studies with preschool children have shown that fathers’ insensitive care is related to concurrent assessments of externalizing symptoms directly (DeKlyen et al., 1998), as well as indirectly, *via* the child’s attachment relationship (Bureau et al., 2017). However, few studies are longitudinal, following children over time to ascertain the lasting effects of paternal sensitivity on their adjustment, and even fewer have assessed paternal sensitivity during the first 2 years of life. Specifically, Rodrigues et al. (2021) recently conducted a meta-analysis of the relation between either paternal sensitivity or father-child attachment security and children’s externalizing behaviors or ADHD symptoms. Of the 14 published studies included in the meta-analysis, there were only three longitudinal studies examining relations between father sensitivity and children’s externalizing behavior. Further, only one study assessed father sensitivity in children under the age of 3, and, in that study, all of the children had an alcoholic father (Eiden et al., 2007). Hence, little is known about how paternal sensitivity during infancy and toddlerhood might affect children’s later development of externalizing symptoms in the general population. Since father sensitivity has been associated with externalizing behavior in older children, it is particularly important to identify whether and how insensitive father-infant interaction relates to children’s later development of externalizing problems. This way, interventions can begin early, before insensitive father-child interactions become habitual and before infants can be negatively affected by insensitive care.

### Father Caregiving and Competitive Coparenting

Low paternal sensitivity may also affect children’s later development of externalizing problems by contributing to more competitive, undermining patterns of coparenting, which could, in turn, promote the development of children’s externalizing symptoms. Coparenting has been examined as the degree to which parents support or undermine each other’s childrearing efforts while working together to care for their children (McConnell and Kerig, 2002). In cooperative coparenting, parents support and assist one another in advancing each other’s parenting efforts. In contrast, competitive coparenting involves the parents undermining or criticizing their partners’ parenting in the presence of their child, jockeying for control of the child, or trying to be the “favorite” parent (McHale et al., 2001).

According to family systems theory, subsystems within the family are interdependent (Minuchin, 1974; Jacobvitz et al., 1999); thus, the quality of dyadic parent-child interactions affects triadic mother-father-child family dynamics, including coparenting quality. The coparenting relationship has been shown to influence father sensitivity with infants at 3.5 months (Brown et al., 2010), but the contribution of paternal sensitivity to coparenting in the mother-father-child triad is less clear. Bernier et al. (2021) found that father-child play at age 4 that was characterized by harmonious communication and mutual cooperation and low emotional ambivalence predicted coparenting quality at age 6. Specifically, these parents engaged in more harmonious and positive exchanges, marked by greater agreement and fewer critical comments and competitive interactions about how to handle the child. These couples also displayed more enjoyment of their child. Further, the interaction of child–mother and child–father attachment security during the preschool years, which is known to be related to parenting sensitivity, significantly predicted the quality of the coparenting relationship (Bureau et al., 2021). Perhaps when fathers are more sensitive, their spouses are more supportive and less likely to undermine the father-child relationship. As a result, the parents may engage in more supportive coparenting, working together cooperatively rather than competitively in caring for their child. Indeed, mothers’ support of fathers’ coparenting decisions has been linked to more cooperative coparenting (Murphy et al., 2017b).

In contrast, mothers may be more critical and undermining of fathers who are insensitive with their child. When mothers are not confident that their spouses are involved and competent caregivers, they are more likely to engage in maternal gatekeeping, defined as maternal attitudes and actions that negatively affect the quality of fathers’ relationship and involvement with their child (Allen and Hawkins, 1999). Maternal gatekeeping often reduces fathers’ involvement in infant care, which further erodes fathers’ caregiving competence (Altenburger et al., 2018). Indeed, mothers’ discouragement and criticism of fathers’ involvement in infant care predicts parents’ reports of poorer coparenting quality (Schoppe-Sullivan et al., 2008).

### Competitive Coparenting and Children’s Externalizing Problems

Numerous studies have found competitive coparenting to be a robust predictor of children’s externalizing problems (Schoppe et al., 2001; Teubert and Pinquart, 2010; Murphy et al., 2016). Competitive coparenting is characterized by parents putting the child in the middle of their coparenting conflicts by undermining each other in front of the child, jockeying for control of the child, and trying to get the child to take sides (McHale et al., 2001). Thus, it necessarily involves triangulation of the child such that the child is put in a position of having to choose between their parents. A meta-analysis of associations between coparenting quality and children’s externalizing behaviors (Teubert and Pinquart, 2010) indicated that children’s externalizing behaviors were positively associated with competitive coparenting (triangulation of the child) and coparenting conflict (parental disagreements about coparenting), and negatively associated with cooperative coparenting. Negative types of coparenting, including competitive coparenting, conflictual coparenting, and low levels of cooperative coparenting often co-occur, making it difficult to determine which of these aspects of negative coparenting contribute to the development of children’s externalizing behaviors (Margolin et al., 2001). It may be that children model the high levels of family conflict they observe during coparenting conflict, which then contributes to their later development of externalizing behaviors (Teubert and Pinquart, 2010). Alternatively, the emotional security hypothesis (Davies and Martin, 2013) postulates that triangulation of the child, the key characteristic of competitive coparenting, may be particularly emotionally threatening to the child, resulting in increased emotional dysregulation, impulsivity, and attention problems. This may, in turn, contribute to the later development of aggression and externalizing problems (Machado and Mosmann, 2020). This has been confirmed in recent studies that found strong associations between competitive coparenting, characterized by triangulation of the child, and children’s later development of externalizing problems from early to middle childhood (Murphy et al., 2016) and from middle childhood to adolescence (Riina and McHale, 2014; Machado and Mosmann, 2020). Moreover, when competitive coparenting, coparenting conflict, negative affect in coparenting, and low cooperative coparenting were simultaneously entered into a model to predict children’s development of externalizing symptoms in middle childhood, only competitive coparenting remained as a significant predictor of externalizing symptoms (Murphy et al., 2016). Thus, in the current study, we focus particularly on competitive coparenting as a consequence of fathers’ less sensitive caregiving and as a predictor of children’s later externalizing symptoms.

### The Current Study

The goal of the present study is to examine multiple pathways from paternal sensitivity in infancy to externalizing behavior in middle childhood. We hypothesize that: (1) father sensitivity will be stable from 8 to 24 months, and father sensitivity at 24 months will predict children’s externalizing symptoms at 7 years; and (2) father sensitivity at 8 months will predict competitive coparenting at 24 months, and competitive coparenting will, in turn, predict children’s externalizing symptoms at age 7. That is, the relation of fathers’ sensitivity at 8 months to externalizing behavior in middle childhood will be mediated by fathers’ continued sensitivity at 24 months and by couples’ competitive coparenting at 24 months.

In our model, we controlled for fathers’ age, education, and family income, since older fathers, those with more education, and those from higher socioeconomic backgrounds tend to engage in more sensitive care with their infants (e.g., Rockville, 2000). We controlled for paternal involvement, since the amount of time fathers spend interacting with their infants has been found to be associated with both father sensitivity and children’s later outcomes [National Institute of Child Health and Human Development (NICHD), 2000]. We also controlled for paternal depression, since parental depression has been associated with lower quality caregiving (e.g., Bronte-Tinkew et al., 2007). In addition, we controlled for child sex because boys are more likely to show externalizing behaviors (Bongers et al., 2004). We also controlled for infant temperament, since it has been associated with parenting quality (Bates et al., 2012), externalizing behaviors (Bradley and Corwyn, 2008), and negative parenting behaviors, such as undermining (Cook et al., 2009). We also controlled for marital satisfaction, since it has been associated with coparenting quality (Schoppe-Sullivan et al., 2004; Christopher et al., 2015). Finally, we controlled for maternal sensitivity, since mother and father sensitivity have been found to be related in previous studies (Barnett et al., 2008).

## Methods

### Participants

Participants were part of a longitudinal study following 125 families over the transition to first-time parenthood, from shortly before they expected their first child until the child was 7 years old (Jacobvitz et al., 2004). Couples were recruited during pregnancy through childbirth classes, public service radio announcements, and flyers distributed at local maternity stores and obstetricians’ offices in a large southwestern United States city. To be eligible for the study, all couples had to be either married (91%) or living together at the start of the study and expecting their first child. Participants were primarily middle class but varied in income level. One-third were at or below poverty level and two-thirds were from middle class backgrounds based on the census data in the mid-1990s when the sample was recruited: 25.6% reported over $60,000 in total family income, 26.4% reported $45,001–$60,000, 24.8% reported $30,001– $45,000, and 23.2% reported their total family income equal to or less than $30,000, which was considered below poverty level. The mean age of mothers was 29.48 (*SD* = 4.73), with a range from 16 to 41 years old and the mean age of fathers was 31.66 (*SD* = 6.17), ranging from 19 to 51 years old. Participants were predominantly White (86% of fathers and 83% of mothers). Other participants identified themselves as Hispanic (10% of fathers and 7% of mothers), and African American (4% of fathers and 2% of mothers). The remaining 12 mothers chose “Other,” and two of them wrote in an ethnicity (Middle Eastern and Indian). Each parent reported their highest level of education. Participants were generally well-educated with 9% of the mothers and 8% of the fathers reporting their highest education level was high school, 25% of the mothers and 34% of the fathers had some training beyond high school but did not graduate from college, 46% of the mothers and 38% of the fathers earned a bachelor’s degree, and 18% of the mothers and 17% of the fathers had a graduate or post college degree. All infants (41% female) were born full-term and none were admitted to the Neonatal Intensive Care Unit. Following each phase of data collection, families received compensation in the form of savings bonds, newsletters, and gifts for their child.

Data from 119 families included paternal sensitivity in infancy. 108 families remained when the children were 24 months, and teacher-reported data on children was available for 71 children when the children were 7 years old. Couples left the study due to moving away, divorce, being too busy to participate, or losing contact with the researchers. Fathers of families who remained in the study for all waves were older (Time 1 *M_*age*_* = 33.09, *SD* = 6.16) than those who did not complete all waves of data collection [Time 1 *M_*age*_* = 30.04, *SD* = 6.12; *t*(121) = 2.75, *p* = 0.007]; thus, we controlled for paternal age in the model. There were no other significant differences by attrition for any of the study variables or demographic variables (i.e., paternal education and family income).

### Procedure

Data were collected in four waves: the first wave took place when couples were expecting their first child, the second wave took place when the child was 8 months old, the third wave at 24 months, and the fourth wave at 7 years of age. Mothers and fathers completed a background information survey to ascertain age, education and income during the first visit. At 8 months, mothers and fathers were independently observed at home playing with and feeding their infants. Mother and father order was counterbalanced. At this visit, mothers and fathers also reported how much time they spent with their infants and they completed a questionnaire that assessed depressive symptoms experienced in the previous week. When the children were 24 months old, mother, father, and child were videotaped at home interacting for 25-min across a series of triadic interaction tasks. During this visit, parents also completed a questionnaire to examine their marital satisfaction. When children were 7 years of age, the children’s teachers completed a questionnaire to assess externalizing symptoms.

### Measures

#### Caregiver Sensitivity (8 Months)

When infants were 8 months old, mothers and fathers were individually videotaped at home during 30-min interactions as they changed their children’s clothes, fed them, and engaged in free-play. Parents were asked to play with their child as they normally would. Mother-infant and father-infant interactions were later coded using the Infant Caregiving Scales (ICS; Hazen, 1997). The ICS consists of 90 items derived from descriptions of caregiving that are provided in the instructions for rating Ainsworth’s three global scales for assessing sensitivity vs. insensitivity, acceptance vs. rejection, and cooperation vs. interference (Ainsworth et al., 1978). The ICS was developed in order to assess sensitive caregiving, as conceptualized by Ainsworth, using a more robust scale consisting of multiple items rather than a single global sensitivity item. Multi-item scales are considered to provide better content validity for assessing abstract constructs compared to single-item scales, as they describe the construct in multiple ways (McIver and Carmines, 1981). They are also more sensitive, having more points of discrimination, and they provide a means of assessing internal consistency of the scale. The sensitivity scale for the ICS, as well as other caregiving scales, including hostile, disengaged, interfering, and role-reversed caregiving, were developed using a criterion sort method (Waters and Deane, 1985), in which expert judges rated each of the 90 items on the ICS based on the extent to which they were diagnostic of each construct. Only the sensitivity scale, which examined the extent to which parents responded promptly and appropriately to their infants’ wishes, was used in the present study. The sensitivity scale consisted of 17 items that the criterion sorters agreed were highly diagnostic of sensitivity or insensitivity (reverse scored). Example items include: “Parent responds to baby when he or she cries,” “Parent’s actions are finely tuned to the baby’s wishes,” “Parent frequently misinterprets baby’s cues; does not seem to understand baby’s nonverbal communication” (reverse scored), and “Parent’s responses are contingent with child’s cues.”

Five coders were trained by observing and coding 14% of the study videotapes as a group with the guidance of the developer of the ICS until they came to a consensus. Then the five trained coders rated mothers and fathers on all ICS items, and 86% of the videotapes were then double coded for reliability. Seven tapes that demonstrated low inter-rater reliability were also rated by a third trained coder. Inter-rater reliability across all items was 0.64. Cronbach’s alpha for the sensitivity subscale was 0.94. Scores averaged across coders were used for data analysis. Construct validity for the sensitivity scale of the ICS was obtained by correlating average scores for ICS sensitivity with the global single-item sensitivity ratings previous obtained from another team of raters who previously rated the same videotapes using the Ainsworth scales; *r*(113) = 0.81, *p* < 0.001. Evidence for concurrent and criterion validity was obtained in later published studies that found that: (1) parents with secure working models of attachment had higher scores on ICS sensitivity than those with insecure working models (McFarland et al., 2012; Poulsen et al., 2019), (2) more positive and less negative affect in prenatal marital interactions predicted mothers’ and fathers’ ICS sensitivity with their infant at 8 months (Poulsen et al., 2019), and (3) parents’ lower ICS sensitivity at 8 months predicted their children’s greater emotional dysregulation as toddlers (Hazen et al., 2010).

#### Caregiver Sensitivity (24 Months)

At 24 months, mothers, fathers, and children came to the university laboratory. Mother-child and father-child interactions were independently videotaped during 20 min of free play and 5 min of clean-up. Next, parent-child dyads completed four problem-solving tasks. The parent was told to let the child first work on the problem independently, then to give “any help you think he/she needs.” The first two problems were easy for the child and involved removing a lure from a space between two closely spaced wooden panels or a tube using a stick. The third task was more difficult. The child was asked to put bristle blocks end to end to remove a lure from a long tube. The final task was beyond the child’s ability, requiring the parent to help the child. This task required the child to weigh down one end of a lever with a block to raise the other end of the level whereby a treat could be reached through a hole in a Plexiglas box. The order in which mothers and fathers interacted with their toddlers was randomized and counterbalanced.

The Infant Caregiving Scales used to code parent interaction with infants was adapted to use for parent interactions with toddlers, creating the 90-item Toddler Caregiving Scales (TCS). A few items were changed so that they were age-appropriate for toddlers (for example, items referring to infant feeding were changed to apply to parent-toddler interaction in teaching tasks), but most were the same except that the word “baby” was replaced by “toddler” or “child.” Items in the toddler sensitivity scale did not include any of the reworded items, but instead included 14 of the original 17 items that comprised the infant sensitivity scale; three were removed because they reduced the overall coefficient alpha. The removed items were: “Parent’s vocalizations to the child are overstimulating (reverse coded),” “Parent provides a voice for child’s wishes,” and “Parent tries to empower and affirm child’s wishes.” These items may be less developmentally appropriate measures of parenting sensitivity with toddlers, especially the second two, which involve the parent speaking for the child. In toddlerhood, sensitive parents seem more likely to speak *to* the child rather than to speak *for* the child.

All 90 items on the TCS were coded by trained coders and 70% of the videotapes were double-coded. Inter-rater reliability was 0.71 for mother sensitivity and 0.72 for father sensitivity. Cronbach’s alpha was 0.94 for mother sensitivity and 0.93 for father sensitivity. At both 8 and 24 months, the average of both coders’ ratings were used for tapes rated by more than one coder. A different set of coders rated parent-child interactions at 8 months and 24 months and coders had no knowledge of scores on any of the other measures.

#### Coparenting Behavior

When children were 24 months old, families (i.e., mother, father, and child) were videotaped in their homes engaging in several triadic interaction tasks. Triadic interactions lasted a total of 25 min. Parents were tasked with a card sorting activity while concurrently working to prepare a snack and change their child’s clothes. These tasks were designed to examine coparenting interactions that forced parents to work on an adult task while simultaneously caring for their child. Parents had 25 min to complete all of the tasks in any order they choose. The time constraint was intended to put mild pressure on the parents. If parents completed the tasks early, they were asked to engage their child in a challenging peg-sorting task.

The interactions were later coded using the Coparenting and Family Rating Scale (CFRS; McHale et al., 2001), informed by structural family theory (Minuchin, 1974). Concurrent, predictive and discriminant validity and test-retest reliability of the scale are well established by McHale et al. (2001) (e.g., McHale et al., 2001; McConnell and Kerig, 2002). Only the Competitive Coparenting scale was used in the present study. Competitive coparenting is defined as the degree to which parents put the child in the middle of their disagreements or undermine or contradict each other in the presence of the child often with the purpose of gaining attention or favoritism from the child. A score of five indicates that parents demonstrated excessive levels of competitive behaviors and no self-awareness. Alternatively, a score of 1 indicated that parents did not demonstrate competitive or undermining behaviors. In addition, if coparenting was nonexistent, for example, if one parent made all the parenting decisions and the other parent went along with them, then a score of 1 was given. Two coders were trained independently and blind to all other data. For scores that differed by more than one point between the coders, the coding team decided on the final ratings. Intraclass correlation was 0.81.

#### Children’s Externalizing Behaviors

When children were 7 years old, each of their teachers completed the Teacher’s Report Form (TRF; Achenbach, 1991). The TRF is composed of 116 items that measure emotional and behavioral problems in the school setting. Teachers rated each item as 0 = “not true,” 1 = “somewhat or sometimes true,” or 2 = “very true or often true.” The current study utilized the externalizing subscale on the TRF, which includes items assessing aggressive and rule-breaking behavior. Inter-rater reliability and test-retest reliability for the TRF are high, with intraclass correlations being in the .90s (Achenbach, 1991).

### Control Variables

#### Fathers’ Involvement in Infant Caregiving

At 8 months postpartum, mothers and fathers reported how much time each parent spent caring for their infant in a typical week. On a chart that covered a week, they independently identified how many hours each parent had spent caring for their child every day from 6 a.m. to 11 p.m. the prior week. To calculate fathers’ share of childcare, mothers and fathers’ scores were averaged and then the percent time that the father spent caring for the child was calculated based on the total number of hours in the week.

#### Paternal Depressive Symptoms

At 8 months postpartum, fathers completed the Center for Epidemiological Studies Depression Scale (CES-D; Radloff, 1977). The CES-D is a 20-item self-report questionnaire in which participants rate how often in the previous week they experienced the depressive symptoms in each statement. Sample items of this measure include, “I felt depressed” and “I thought my life had become a failure.” Each item was rated on a 4-point Likert scale ranging from “Rarely or none of the time” to “Most or all of the time.” Participants’ total item scores were combined to represent the general depression that they experienced the previous week. The CES-D has established validity, adequate test-retest reliability, and high internal consistency (Radloff, 1977).

#### Marital Satisfaction

At 24 months postpartum, fathers completed the Marital Opinion Questionnaire (MOQ; Huston and Vangelisti, 1991). The MOQ encompasses two parts that examine mothers and fathers’ relational happiness throughout the previous 2 months. In the first part, mothers and fathers rated ten bipolar adjectives (e.g., miserable-enjoyable, rewarding-disappointing) on a 7-point semantic differential scale. In the second part, mothers and fathers rated a single item that assessed their overall satisfaction with their marriage. This item was rated on a 7-point scale. When creating a marital satisfaction variable, adjective pairs (i.e., free-tied down and hard-easy) were excluded because they were not correlated with the other adjective pairs. The average of the remaining eight bipolar adjectives was calculated. Internal consistency of the eight adjectives was high for both mothers and fathers (from 0.90 to 0.94). Because the scores from the eight bipolar adjectives and the single item were highly correlated to each other (from 0.53 to 0.77), these scores were then averaged to constitute the marital satisfaction variable for each participant. According to Huston and Vangelisti (1991), the MOQ is highly correlated with similar established measures of marital satisfaction, such as the satisfaction subscale from Spanier’s Dyadic Adjustment Scale (Spanier, 1976).

#### Infant Temperament

At 3 to 6 weeks postpartum, mothers completed the Infant Behavior Questionnaire (IBQ; Rothbart, 1981). The IBQ uses 87 items to measure infant temperament on the following six domains: infants’ activity level, smiling and laughter, fear, distress to limitations, soothability, and duration of orienting. The frequency of behaviors for each domain was rated on a 7-point scale from 1 = never to 7 = always, with higher scores indicating higher reactivity. Smiling and laughter, activity level, and duration of orienting comprise the positive reactivity scales, whereas fear and distress to limitations comprise the negative reactivity scales. Following Rothbart (1986) suggestion, the current study utilizes a composite net negative reactivity scale that was created by subtracting the standardized positive reactivity scales from the standardized negative reactivity scales. We examined net negative reactivity, rather than using negative reactivity alone, because we assumed that the extent to which infant temperament would affect parental caregiving or children’s later development of externalizing behavior would be a function of the child’s temperament as a whole, such that the child’s positive reactivity would mitigate the effects of their negative reactivity. The composite net negative reactivity measure had a Cronbach’s alpha of 0.77. The reliability and validity of this scale is well established (Rothbart, 1986).

#### Family Income

When couples were expecting their first children, parents individually reported their education and age. They also reported family income at 8 months, 24 months, and 7 years. Mother and fathers’ reported incomes were then averaged to create a composite family income variable.

## Results

### Overview of Analyses

**FIGURE 1:**
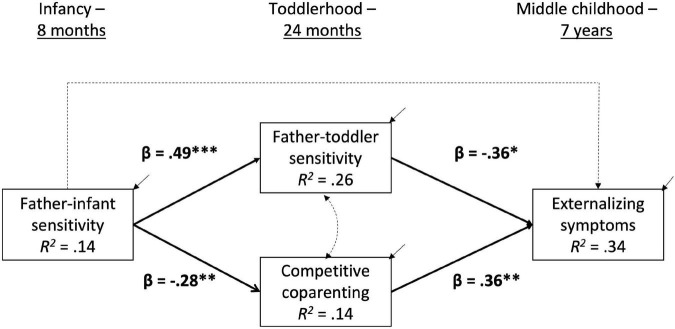
Structural model of father sensitivity, coparenting, and child externalizing symptoms.

We conducted path analyses in a structural equation modeling framework using Mplus 7.4 (Muthén and Muthén, 1998-2012). All variables used met the requirements for normality; thus, we used ML estimation to analyze the models. We addressed the missing data from this longitudinal study through full-information maximum likelihood (FIML). This method allows all available data to contribute to parameter estimation but does not impute any missing values (Enders and Bandalos, 2001; Mueller and Hancock, 2018). The effects of paternal sensitivity with their infants were modeled on paternal sensitivity during toddlerhood and dyadic competitive coparenting (see Figure 1), which were then modeled on child externalizing problems. The effects of maternal sensitivity, paternal age, paternal depressive symptoms, family income, child sex, child temperament, division of childcare, parental education, and paternal marital satisfaction were all accounted for within the model. The model fit was acceptable: χ^2^ (32) = 39.56, *p* = 0.168; RMSEA = 0.04 (0.00–0.09); CFI = 0.88; SRMR = 0.05.

### Preliminary Analyses

Paternal sensitivity was significantly linked across time (*r* = 0.49, *p* < 0.001), such that fathers who were more sensitive with their infants at 8 months were also more sensitive with their toddlers at 24 months. Paternal sensitivity at 8 months (but not 24 months) was also significantly related to lower levels of competitive coparenting in the triadic family interactions at 24 months (*r* = −0.22, *p* = 0.039). Higher levels of paternal sensitivity at 24 months (but not 8 months) were also associated with lower levels of teacher-reported externalizing behaviors when children were school-aged (*r* = −0.30, *p* = 0.025). Finally, higher levels of competitive coparenting were related to higher levels of externalizing behaviors (*r* = 0.41, *p* = 0.002). See Table 1 for all correlations and descriptive statistics.

**TABLE 1:** Bivariate correlations of study variables.

	Variable	1	2	3	4	5	6	7	8	9	10	11	12	13	14	15	16
1	Father sensitivity, 8 month.	−															
2	Father sensitivity, 24 month.	0.49***	−														
3	Competitive coparenting	−0.22*	–0.16	−													
4	TRF: Externalizing	–0.13	−0.30*	−0.41**	−												
5	Father Involvement	–0.03	0.09	–0.12	–0.14	−											
6	Child sex	0.04	0.16	–0.00	–0.20	–0.08	−										
7	Father age	0.23*	0.15	–0.04	–0.14	0.01	0.02	−									
8	Father education	0.20*	0.25*	0.06	0.03	0.00	0.00	0.32***	−								
9	Father depression	0.14	0.12	–0.02	0.01	0.18	–0.13	–0.10	–0.12	−							
10	Mother sensitivity, 8 month.	0.25**	0.16	–0.09	–0.10	–0.00	–0.07	0.16	0.22*	–0.02	−						
11	Mother sensitivity, 24 month.	0.01	0.03	–0.11	–0.13	–0.17	0.08	0.02	–0.11	0.11	–0.02	−					
12	Father marital satisfactn	−0.23*	–0.18	–0.11	–0.06	–0.09	0.02	–0.08	–0.09	−0.23*	0.09	–0.04	−				
13	Family income, 8 month.	0.12	0.20	−0.29**	–0.10	–0.06	0.06	0.38***	0.35***	–0.10	0.13	0.03	–0.09	−			
14	Family income, 24 month.	–0.05	0.06	–0.05	–0.05	0.06	0.05	0.23*	0.25*	–0.09	–0.00	0.13	–0.13	0.80***	−		
15	Family income, 7 years.	0.18	0.19	–0.02	–0.03	0.01	0.12	–0.01	0.18	–0.04	–0.11	–0.09	–0.21	0.49***	0.62***	–	
16	Infant temperament	–0.13	–0.12	–0.02	–0.12	0.15	0.07	−0.23*	–0.18	0.02	0.10	–0.05	–0.05	–0.07	0.03	0.04	–
	*Mean*	4.44	5.26	1.79	51.34	0.34	0.41	31.67	4.53	7.76	4.41	5.60	5.74	3.50	3.81	4.21	0.49
	*(SD)*	(0.83)	(1.02)	(0.91)	(8.61)	(0.12)	(0.49)	(6.31)	(1.13)	(5.94)	(0.90)	(0.92)	(1.04)	(1.20)	(1.10)	(0.85)	(1.68)

*N = 125.*

*For child sex, female is the reference group.*

TRF, Teacher Report Form; *p < 0.05, **p < 0.01, ***p < 0.001.

*Significant correlations are shown in bold.*

### Model of Paternal Sensitivity, Coparenting, and Child Externalizing Symptoms

In the full structural equation model shown in Figure 1, paternal sensitivity during infancy had a direct effect on parental sensitivity and coparenting quality in toddlerhood, such that fathers who were more sensitive with their infants were more likely to be sensitive with their toddlers (β = 0.49, *p* < 0.001) and exhibited lower levels of competitive coparenting (β = −0.28, *p* = 0.005).

Paternal sensitivity during infancy did not have a direct effect on child externalizing symptoms at age 7 (β = 0.07, *p* = 0.57). However, paternal sensitivity during toddlerhood did have a direct effect on child externalizing symptoms (β = −0.36, *p* = 0.019); children whose fathers had been more sensitive exhibited fewer externalizing behaviors. Competitive coparenting during toddlerhood was also significantly linked to later externalizing behaviors (β = 0.36, *p* = 0.003); parents who engaged in more competitive coparenting were more likely to have a child who later demonstrated externalizing behaviors.

#### Indirect Effects

Indirect effects were calculated using the delta method, which utilizes the standard errors of each pathway and the covariance between the two (Bollen, 1989). Father-toddler sensitivity had an indirect effect on the relation between father-infant sensitivity and child externalizing behaviors (β = −0.17, *p* = 0.031). Competitive coparenting also had a significant indirect effect on the relation between father-infant sensitivity and child externalizing behaviors (β = −0.10, *p* = 0.049).

#### Covariates

All covariates (concurrent maternal sensitivity, fathers’ marital satisfaction, paternal age, paternal education, paternal depressive symptoms, concurrent household income, child sex, division of childcare, and temperament) were included in the model based on theory and previous research, as shown in Figure 1. Paternal sensitivity during infancy was significantly related to maternal sensitivity during infancy (β = 0.21, *p* = 0.019) and concurrent paternal depression (β = 0.17, *p* = 0.046). Higher levels of competitive coparenting were linked to lower paternal marital satisfaction during toddlerhood (β = −0.20, *p* = 0.042). All other covariates were not statistically significant when considered simultaneously in the full model.

## Discussion

This prospective longitudinal study following families over 6 years identifies early risk factors for childhood psychopathology. Findings in this study underscore the unique role of sensitivity in father-child interactions during the first 2 years on children’s well-being in middle childhood. We found stability in the level of fathers’ sensitive care with their child from 8 to 24 months, and we identified two different indirect pathways from fathers’ insensitive interactions at 8 months to their children’s externalizing problems at age 7. First, fathers’ sensitivity at 8 months in the context of caregiving activities (feeding, clothes change) and play predicted externalizing problems in middle childhood through fathers sensitivity at 24 months. Secondly, fathers’ sensitivity at 8 months also significantly predicted externalizing problems in middle childhood through competitive, undermining coparenting interactions at 24 months.

Finding that the quality of early paternal care plays an important role in children’s later adjustment is consistent with previous research showing that insensitive and intrusive control, and harsh, coercive, and punitive parenting are strongly implicated in the development and stability of conduct disorders. In contrast, warmth, responsiveness and sensitivity are associated with lower rates of later behavior problems (Campbell et al., 2000; Trautmann-Villalba et al., 2006) and higher rates of prosocial behavior (Hastings et al., 2007; Ferreira et al., 2016).

It is interesting to note that father sensitivity at 8 months did not directly predict children’s externalizing behavior, but it did predict father sensitivity at 24 months. This result is consistent with Towe-Goodman et al. (2014) study demonstrating that paternal sensitivity and support at 24 months, but not 7 months, was associated with children’s executive function at age 3. Our results indicate that continuity of sensitive paternal caregiving from 8 to 24 months is particularly important. Not only do fathers spend more one-on-one time with their children as they get older, but they also engage in more stimulating play, such as rough-and-tumble play (MacDonald and Parke, 1986). In the context of such highly stimulating play, sensitive fathers can comfort and calm an overstimulated, fearful, or angry child, which may help them regulate strong emotions and avoid externalizing behaviors (Paquette, 2004; Hazen et al., 2010). Indeed, a recent meta-analysis found that children’s engagement in stimulating physical play with fathers was related to better social and emotional skills, and higher self-regulation, all of which were negatively related to externalizing problems (Stgeorge and Freeman, 2017).

This study also demonstrated the unique role of sensitive paternal caregiving in the coparenting alliance and children’s later adjustment. Paternal sensitivity was associated with coparenting, which was in turn associated with children’s externalizing behavior, even after controlling for maternal sensitivity at both 8 and 24 months, paternal depression, paternal involvement, and marital satisfaction. Previous studies have examined the effects of undermining coparenting behavior on mothers and fathers caregiving quality (Jia and Schoppe-Sullivan, 2011). Yet, from a family systems perspective it is also important to understand how caregiving quality is associated with the developing coparenting alliance observed in the triadic interactions (Brown et al., 2022).

This study is one of the first to identify the contribution of fathers’ sensitivity with infants during the first year of life to the coparenting relationship with younger children. Our findings are consistent with previous studies with older children showing father-child interaction quality with 18-month-olds was associated with triadic coparenting interactions when children were 6 years old (Bernier et al., 2021). Mothers may undermine their husbands’ input about parenting when they perceive their husbands as incompetent as caregivers, leading to more critical and competitive coparenting behavior. Our findings are also consistent with past studies that have found that attachment security, characterized by sensitive parenting, is related to coparenting quality (Bureau et al., 2021).

This study has several strengths. The study was longitudinal and included observational assessments of dyadic and triadic interactions. Also, fathers were observed interacting with their infants across multiple contexts, including feeding, changing their infants’ clothes, and playing with their infant. Moreover, unlike many studies that rely solely on parent reports of childhood behavior problems, the current study obtained assessments of children’s externalizing symptoms from the children’s teacher. This minimizes the likelihood that the parents’ relationship with their child influenced ratings of their children’s well-being. Further, most previous studies have examined paternal caregiving with older children in the context of play and problem-solving tasks. Findings of this study demonstrate that father-child interaction in infancy can have long-term implications for children’s healthy social-emotional development.

This study also has several limitations. First, we did not assess coparenting soon after the baby was born. It is possible that there is continuity in coparenting quality over the child’s first 2 years of life. It may be the case that undermining coparenting soon after the child’s birth spilled over to fathers’ behavior with the infant, which furthered competitive and undermining coparenting behavior at 24 months. Moreover, the study includes observational data and data collected over 7 years, but the sample is small. There was sufficient statistical power to detect the direct effects, above 0.80, based on a Monte Carlo Simulation that took into account missing data. However, the power to detect indirect effects was lower, ranging from 0.48 to 0.71. It will be important to replicate these findings with a larger sample. Finally, although the sample was mixed socioeconomically, it was primarily white and included only heterosexual two-parent families. It is unclear whether findings in this study generalize to families with non-residential fathers, single fathers, gay couples or parents with different gender orientations.

It may also be important for future studies to consider the role of the marital relationship in the association between the father-infant relationship and the coparenting alliance. Mothers who view their husbands’ caregiving more positively may be more likely to engage and support fathers in caring for their children, contributing to warmth, support and positivity in the marriage. At the same time, when the quality of the marriage declines, fathers may become less involved in caregiving (Christopher et al., 2015), compromising the quality of care they provide their children (Murphy et al., 2017b).

Findings in this study highlight the importance of developing effective early interventions to help fathers be more sensitive, responsive, and emotionally available to their infants, when needed, and to engage in less interfering and intrusive behavior. Fostering paternal sensitivity early in children’s lives could help improve the developing coparenting relationship. These findings also suggest that it is important to strengthen both the father-child dyadic and the mother-father-toddler triadic coparenting relationships. This can reduce the likelihood that children will engage in aggressive and rule breaking behavior in school at a time when learning appropriate social skills and making friends is critical to their well-being.

## Data Availability Statement

The raw data supporting the conclusions of this article will be made available by the authors, without undue reservation.

## Ethics Statement

The studies involving human participants were reviewed and approved by the Internal Review Board (IRB) at the University of Texas at Austin. Written informed consent to participate in this study was provided by the participants’ legal guardian/next of kin.

## Author Contributions

DJ wrote the initial draft of the manuscript, contributed to the conceptualization, organized the data collection, and revised the manuscript. NH contributed to the conceptualization, organized data collection, organized coding of the data, wrote parts of the manuscript, and assisted with revisions. AA contributed to the conceptualization, performed the statistical analyses, wrote the initial draft of the results section, and created the figure and initial versions of the table. GA, ZT, and SZ contributed to the conceptualization, wrote parts of the manuscript, and assisted with revisions. All authors contributed to the article and approved the submitted version.

## Conflict of Interest

The authors declare that the research was conducted in the absence of any commercial or financial relationships that could be construed as a potential conflict of interest.

## Publisher’s Note

All claims expressed in this article are solely those of the authors and do not necessarily represent those of their affiliated organizations, or those of the publisher, the editors and the reviewers. Any product that may be evaluated in this article, or claim that may be made by its manufacturer, is not guaranteed or endorsed by the publisher.

## References

[B1] Achenbach T. M. (1991). *Manual for the Teacher’s Report From and 1991 Profile.* Burlington, VT: University of Vermont, Department of Psychiatry.

[B2] Ainsworth M. D. S. Blehar M. C. Waters E. Wall S. (1978). *Patterns of Attachment: A Psychological Study Of The Strange Situation.* Mahwah, NJ: Lawrence Erlbaum.

[B3] Allen S. M. Hawkins A. J. (1999). Maternal gatekeeping: mothers’ beliefs and behaviors that inhibit greater father involvement in family work. *J. Marriage Fam.* 61 199–212. 10.2307/353894

[B4] Altenburger L. E. Schoppe-Sullivan S. J. Dush C. M. K. (2018). Associations between maternal gatekeeping and fathers’ parenting quality. *J. Child Fam. Stud.* 27 2678–2689. 10.1007/s10826-018-1107-3

[B5] Bakermans-Kranenburg M. J. Lotz A. Alyousefi-van Dijk K. van IJzendoorn M. (2019). Birth of a father: fathering in the first 1,000 days. *Child Dev. Perspect.* 13 247–253. 10.1111/cdep.12347 31894183 PMC6919930

[B6] Barnett M. A. Deng M. Mills-Koonce W. R. Willoughby M. Cox M. (2008). Interdependence of parenting of mothers and fathers of infants. *J. Fam. Psychol.* 22 561–573. 10.1037/0893-3200.22.3.561 18729670

[B7] Bates J. E. Schermerhorn A. C. Petersen I. T. (2012). Temperament and parenting in developmental perspective. *Handb. Temperament* 425–441.

[B8] Behrens K. Y. Hart S. L. Parker S. (2012). Maternal sensitivity: evidence of stability across time, contexts, and measurement instruments. *Infant Child Dev.* 21 348–355. 10.1002/icd.1747

[B9] Bernier A. Cyr C. Matte-Gagné C. Tarabulsy G. M. (2021). Parent–child interactions as predictors of coparenting: a longitudinal study of family subsystems. *J. Fam. Stud.* 1–16. 10.1080/13229400.2021.1908909

[B10] Bollen K. A. (1989). A new incremental fit index for general structural equation models. *Sociol. Methods Res.* 17 303–316. 10.1177/0049124189017003004

[B11] Bongers I. L. Koot H. M. Van Der Ende J. Verhulst F. C. (2004). Developmental trajectories of externalizing behaviors in childhood and adolescence. *Child Dev.* 75 1523–1537. 10.1111/j.1467-8624.2004.00755.x 15369529

[B12] Bradley R. H. Corwyn R. F. (2008). Infant temperament, parenting, and externalizing behavior in first grade: a test of the differential susceptibility hypothesis. *J. Child Psychol. Psychiatry* 49 124–131. 10.1111/j.1469-7610.2007.01829.x 18211274

[B13] Bronte-Tinkew J. Moore K. A. Matthews G. Carrano J. (2007). Symptoms of major depression in a sample of fathers of infants: sociodemographic correlates and links to father involvement. *J. Fam. Issues* 28 61–99. 10.1177/0192513X06293609

[B14] Brown G. L. Mangelsdorf S. C. Neff C. Shigeto A. Aytuglu A. Thomas C. R. (2022). Infant attachment configurations with mothers and fathers: implications for triadic interaction quality and children’s parental preferences. *Early Child. Res. Q.* 58, 155–164. 10.1016/j.ecresq.2021.09.004

[B15] Brown G. L. Schoppe-Sullivan S. J. Mangelsdorf S. C. Neff C. (2010). Observed and reported supportive coparenting as predictors of infant–mother and infant–father attachment security. *Early Child Dev. Care* 180 121–137. 10.1080/03004430903415015 25983376 PMC4430853

[B16] Bureau J. F. Deneault A. A. Yurkowski K. Martin J. Quan J. Sezlik S. (2021). The interaction of child–father attachment and child–mother attachment in the prediction of observed coparenting. *Psychol. Men Masc.* 22 512–520. 10.1037/men0000309

[B17] Bureau J. Martin J. Yurkowski K. Schmiedel S. Quan J. Moss E. (2017). Correlates of child–father and child–mother attachment in the preschool years. *Attach. Hum. Dev.* 19 130–150. 10.1080/14616734.2016.1263350 27899058

[B18] Cabrera N. Volling B. Barr R. (2018). Fathers are parents, too! Widening the lens on parenting for children’s development. *Child Dev. Perspect.* 12 152–157. 10.1111/cdep.12275

[B19] Campbell S. B. Shaw D. S. Gilliom M. (2000). Early externalizing behavior problems: toddlers and preschoolers at risk for later maladjustment. *Dev. Psychopathol.* 12 467–488. 10.1017/S0954579400003114 11014748

[B20] Christopher C. Umemura T. Mann T. Jacobvitz D. Hazen N. (2015). Marital quality over the transition to parenthood as a predictor of coparenting. *J. Child Fam. Stud.* 24 3636–3651. 10.1007/s10826-015-0172-0

[B21] Cook J. C. Schoppe-Sullivan S. J. Buckley C. K. Davis E. F. (2009). Are some children harder to coparent than others? Children’s negative emotionality and coparenting relationship quality. *J. Fam. Psychol.* 23 606–610. 10.1037/a0015992 19685995 PMC3150515

[B22] Dallaire D. H. Weinraub M. (2005). The stability of parenting behaviors over the first 6 years of life. *Early Child. Res. Q.* 20 201–219. 10.1016/j.ecresq.2005.04.008

[B23] Davies P. T. Martin M. J. (2013). The reformulation of emotional security theory: the role of children’s social defense in developmental psychopathology. *Dev. Psychopathol.* 25 1435–1454. 10.1017/S0954579413000709 24342849 PMC3918896

[B24] DeKlyen M. Biernbaum M. A. Speltz M. L. Greenberg M. T. (1998). Fathers and preschool behavior problems. *Dev. Psychol.* 34 264–275. 10.1037/0012-1649.34.2.264 9541779

[B25] Eiden R. D. Edwards E. P. Leonard K. E. (2007). A conceptual model for the development of externalizing behavior problems among kindergarten children of alcoholic families: role of parenting and children’s self-regulation. *Dev. Psychol.* 43 1187–1201. 10.1037/0012-1649.43.5.1187 17723044 PMC2720575

[B26] Enders C. Bandalos D. (2001). The relative performance of full information maximum likelihood estimation for missing data in structural equation models. *Struct. Equ. Modeling* 8 430–457. 10.1207/S15328007SEM0803_5 33486653

[B27] Feldman R. (2010). The relational basis of adolescent adjustment: trajectories of mother–child interactive behaviors from infancy to adolescence shape adolescents’ adaptation. *Attach. Hum. Dev.* 12 173–192. 10.1080/14616730903282472 20390528

[B28] Ferreira T. Cadima J. Matias M. Vieira J. M. Leal T. Matos P. M. (2016). Preschool children’s prosocial behavior: the role of mother–child, father–child and teacher–child relationships. *J. Child Fam. Stud.* 25 1829–1839. 10.1007/s10826-016-0369-x

[B29] Grossmann K. Grossmann K. E. (2020). Essentials when studying child-father attachment: a fundamental view on safe haven and secure base phenomena. *Attach. Hum. Dev.* 22 9–14. 10.1080/14616734.2019.1589056 30898025

[B30] Hastings P. D. McShane K. E. Parker R. Ladha F. (2007). Ready to make nice: parental socialization of young sons’ and daughters’ prosocial behaviors with peers. *J. Genet. Psychol.* 168 177–200. 10.3200/GNTP.168.2.177-20 17936971

[B31] Hazen N. (1997). *Infant Caregiving Scales. Unpublished Assessment.* Austin, TX: University of Texas at Austin.

[B32] Hazen N. L. McFarland L. Jacobvitz D. Boyd-Soisson E. (2010). Fathers’ frightening behaviors and sensitivity with infants: relations with fathers’ attachment representations, father–infant attachment, and children’s later outcomes. *Early Child Dev. Care* 180 51–69. 10.1080/03004430903414703

[B33] Huston T. L. Vangelisti A. L. (1991). Socioemotional behavior and satisfaction in marital relationships: a longitudinal study. *J. Pers. Soc. Psychol.* 61 721–733. 10.1037/0022-3514.61.5.721 1753328

[B34] Jacobvitz D. Hazen N. Curran M. Hitchens K. (2004). Observations of early triadic family interactions: boundary disturbances in the family predict depressive, anxious, and ADHD symptoms in middle childhood. *Dev. Psychopathol.* 16 577–592. 10.1017/S0954579404004675 15605626

[B35] Jacobvitz D. Morgan E. Kretchmar M. Morgan Y. (1991). The transmission of mother-child boundary disturbances across three generations. *Dev. Psychopathol.* 3 513–527. 10.1017/S0954579400007665

[B36] Jacobvitz D. Riggs S. Johnson E. (1999). “Cross-sex and same-sex family alliances: immediate and long-term effects on sons and daughters,” in *Burdened Children: Theory, Research, And Treatment Of Parentification*, eds Chase N. D. Chase N. D. (Thousand Oaks, CA: Sage Publications, Inc), 34–55. 10.4135/9781452220604.n2

[B37] Jia R. Schoppe-Sullivan S. J. (2011). Relations between coparenting and father involvement in families with preschool-age children. *Dev. Psychol.* 47 106–118. 10.1037/a0020802 21244153 PMC3279926

[B38] Lorber M. F. Egeland B. (2009). Infancy parenting and externalizing psychopathology from childhood through adulthood: developmental trends. *Dev. Psychol.* 45 909–912. 10.1037/a0015675 19586169 PMC4532673

[B39] MacDonald K. Parke R. D. (1986). Parent-child physical play: the effects of sex and age of children and parents. *Sex Roles J. Res.* 15 367–378. 10.1007/BF00287978

[B40] Machado M. R. Mosmann C. P. (2020). Coparental conflict and triangulation, emotion regulation, and externalizing problems in adolescents: direct and indirect relationships. *Paidéia (Ribeirão Preto)* 30:e3004. 10.1590/1982-4327e3004

[B41] Margolin G. Gordis E. B. John R. S. (2001). Coparenting: a link between marital conflict and parenting in two-parent families. *J. Fam. Psychol.* 15 3–21. 10.1037//0893-3200.15.1.3 11322083

[B42] Masten A. S. Roisman G. I. Long J. D. Burt K. B. Obradović J. Riley J. R. (2005). Developmental cascades: linking academic achievement and externalizing and internalizing symptoms over 20 years. *Dev. Psychol.* 41 733–746. 10.1037/0012-1649.41.5.733 16173871

[B43] McConnell M. C. Kerig P. K. (2002). Assessing coparenting in families of school-age children: validation of the coparenting and family rating system. *Can. J. Behav. Sci.* 34 44–58. 10.1037/h0087154

[B44] McFarland L. Hazen N. Jacobvitz D. Boyd-Soisson E. (2012). The development of father-child attachment: associations between adult attachment representations, recollections of childhood experiences and caregiving. *Early Child Dev. Care* 182 701–721. 10.1080/03004430.2011.57307

[B45] McHale J. P. Kuersten-Hogan R. Lauretti A. (2001). “Evaluating coparenting and family-level dynamics during infancy and early childhood: the coparenting and family rating system,” in *Family Observational Coding Systems: Resources for Systemic Research*, eds Kerig P. K. Lindahl K. M. (Mahwah, NJ: Erlbaum), 151–170.

[B46] McIver J. Carmines E. G. (1981). *Unidimensional Scaling*, Vol. 24. Thousand, CA: Sage.

[B47] Minuchin S. (1974). *Families & Family Therapy.* Cambridge: Harvard Univ. Press.

[B48] Mueller R. O. Hancock G. M. (2018). “Structural equation modeling,” in *The Reviewer’s Guide to Quantitative Methods in the Social Sciences*, 2nd Edn, eds Hancock G. M. Stapleton L. M. Mueller R. O. (Abingdon: Routledge), 445–456. 10.4324/9781315755649

[B49] Murphy S. E. Boyd-Soisson E. Jacobvitz D. B. Hazen N. L. (2017a). Dyadic and triadic family interactions as simultaneous predictors of children’s externalizing behaviors. *Fam. Relat.* 66 346–359. 10.1111/fare.12225

[B50] Murphy S. E. Gallegos M. I. Jacobvitz D. B. Hazen N. L. (2017b). Coparenting dynamics: mothers’ and fathers’ differential support and involvement. *Pers. Relationsh.* 24 917–932. 10.1111/pere.12221

[B51] Murphy S. E. Jacobvitz D. B. Hazen N. L. (2016). What’s so bad about competitive coparenting? Family-level predictors of children’s externalizing symptoms. *J. Child Fam. Stud.* 25 1684–1690. 10.1007/s10826-015-0321-5

[B52] Muthén L. K. Muthén B. O. (1998-2012). *Mplus User’s Guide*, 7th Edn. Los Angeles, CA: Muthén & Muthén

[B53] National Institute of Child Health and Human Development (NICHD) (2000). Factors associated with fathers’ caregiving activities and sensitivity with young children. *J. Fam. Psychol.* 14 200–219. 10.1037//0893-3200.14.2.200 10870290

[B54] Paquette D. (2004). Theorizing the father-child relationship: mechanisms and developmental outcomes. *Hum. Dev.* 47 193–219. 10.1159/000078723

[B55] Poulsen H. Hazen N. Jacobvitz D. (2019). Parents’ joint attachment representations and caregiving: the moderating role of marital quality. *Attach. Hum. Dev.* 21 597–615. 10.1080/14616734.2018.1492003 29969948

[B56] Radloff L. S. (1977). The CES-D Scale: a self-report depression scale for research in the general population. *Appl. Psychol. Meas.* 1 385–401. 10.1177/014662167700100306 26918431

[B57] Riina E. M. McHale S. M. (2014). Bidirectional influences between dimensions of coparenting and adolescent adjustment. *J. Youth Adolesc.* 43 257–269. 10.1007/s10964-013-9940-6 23539238 PMC3769435

[B58] Rockville M. (2000). Factors associated with fathers’ caregiving activities and sensitivity with young children. *J. Fam. Psychol.* 14 200–219. 10.1037/0893-3200.14.2.200 10870290

[B59] Rodrigues M. Sokolovic N. Madigan S. Luo Y. Silva V. Misra S. (2021). Paternal sensitivity and children’s cognitive and socioemotional outcomes: a meta-analytic review. *Child Dev.* 92 554–577. 10.1111/cdev.13545 33511634

[B60] Rothbart M. K. (1981). Measurement of temperament in infancy. *Child Dev.* 52 569–578. 10.2307/1129176

[B61] Rothbart M. K. (1986). Longitudinal observation of infant temperament. *Dev. Psychol.* 22 356–365. 10.1037/0012-1649.22.3.356

[B62] Schoppe S. J. Mangelsdorf S. C. Frosch C. A. (2001). Coparenting, family process, and family structure: implications for preschoolers’ externalizing behavior problems. *J. Fam. Psychol.* 15 526–545. 10.1037/0893-3200.15.3.526 11584800

[B63] Schoppe-Sullivan S. J. Brown G. L. Cannon E. A. Mangelsdorf S. C. Sokolowski M. S. (2008). Maternal gatekeeping, coparenting quality, and fathering behavior in families with infants. *J. Fam. Psychol.* 22 389–398. 10.1037/0893-3200.22.3.389 18540767

[B64] Schoppe-Sullivan S. J. Mangelsdorf S. C. Frosch C. A. McHale J. L. (2004). Associations between coparenting and marital behavior from infancy to the preschool years. *J. Fam. Psychol.* 18 194–207. 10.1037/0893-3200.18.1.194 14992621

[B65] Spanier G. B. (1976). Measuring dyadic adjustment: new scales for assessing the quality of marriage and similar dyads. *J. Marriage Fam.* 38 15–28. 10.2307/350547

[B66] Stgeorge J. Freeman E. (2017). Measurement of father-child rough-and-tumble play and its relations to child behavior. *Infant Ment. Health J.* 38 709–725. 10.1002/imhj.21676 29088498

[B67] Teubert D. Pinquart M. (2010). The association between coparenting and child adjustment: a meta-analysis. *Parent. Sci. Pract.* 10 286–307. 10.1080/15295192.2010.492040

[B68] Towe-Goodman N. R. Willoughby M. Blair C. Gustafsson H. C. Mills-Koonce W. R. Cox M. J. (2014). Fathers’ sensitive parenting and the development of early executive functioning. *J. Fam. Psychol.* 28 867–876. 10.1037/a0038128 25347539 PMC4261022

[B69] Trautmann-Villalba P. Gschwendt M. Schmidt M. H. Laucht M. (2006). Father–infant interaction patterns as precursors of children’s later externalizing behavior problems. *Eur. Arch. Psychiatry Clin. Neurosci.* 256 344–349. 10.1007/s00406-006-0642-x 16900440

[B70] Waters E. Deane K. E. (1985). Defining and assessing individual differences in attachment relationships: Q-Methodology and the organization of behavior in infancy and early childhood. *Monogr. Soc. Res. Child Dev.* 50 41–65. 10.2307/3333826

